# Hot Melt Extrusion: Highlighting Physicochemical Factors to Be Investigated While Designing and Optimizing a Hot Melt Extrusion Process

**DOI:** 10.3390/pharmaceutics10030089

**Published:** 2018-07-11

**Authors:** Roberta Censi, Maria Rosa Gigliobianco, Cristina Casadidio, Piera Di Martino

**Affiliations:** School of Pharmacy, University of Camerino, Via S. Agostino, 62032 Camerino, Italy; roberta.censi@unicam.it (R.C.); maria.gigliobianco@unicam.it (M.R.G.); cristina.casadidio@unicam.it (C.C.)

**Keywords:** hot-melt extrusion, solid state stability, solid dispersion, thermal methods, X-ray powder diffractometry, spectroscopic techniques, microscopic methods, mechanical analyses, dissolution testing

## Abstract

Hot-melt extrusion (HME) is a well-accepted and extensively studied method for preparing numerous types of drug delivery systems and dosage forms. It offers several advantages: no solvents are required, it is easy to scale up and employ on the industrial level, and, in particular, it offers the possibility of improving drug bioavailability. HME involves the mixing of a drug with one or more excipients, in general polymers and even plasticizers, which can melt, often forming a solid dispersion of the drug in the polymer. The molten mass is extruded and cooled, giving rise to a solid material with designed properties. This process, which can be realized using different kinds of special equipment, may involve modifications in the drug physicochemical properties, such as chemical, thermal and mechanical characteristics thus affecting the drug physicochemical stability and bioavailability. During process optimization, the evaluation of the drug solid state and stability is thus of paramount importance to guarantee stable drug properties for the duration of the drug product shelf life. This manuscript reviews the most important physicochemical factors that should be investigated while designing and optimizing a hot melt extrusion process, and by extension, during the different pre-formulation, formulation and process, and post-formulation phases. It offers a comprehensive evaluation of the chemical and thermal stability of extrudates, the solid physical state of extrudates, possible drug-polymer interactions, the miscibility/solubility of the drug-polymer system, the rheological properties of extrudates, the physicomechanical properties of films produced by hot melt extrusion, and drug particle dissolution from extrudates. It draws upon the last ten years of research, extending inquiry as broadly as possible.

## 1. Introduction

First developed and used in the plastics industry, hot melt extrusion (HME) has been applied in the pharmaceutical industry as a simple, reproducible, and fast method for producing many solid dosage forms of drugs [1,2] for different delivery routes, such as the oral route (granules, pellets, and tablets) [3,4,5,6], the transdermal and transmucosal route [7], and the subcutaneous route (implants) [8,9,10], some of the most widely developed applications are for:

1. Taste masking [11,12]
2. Improved dissolution of poorly soluble drugs [13,14,15,16]
3. Sustained release formulations [17,18] and
4. Preparation of nanosystems [19,20,21,22].

The HME process makes it possible to convert a mix of raw materials into a product with specific characteristics, such as uniform shape and density, by forcing the mix through a die under controlled conditions [23]. For this, HME exploits a molten system, the viscosity of which must be controlled to enable the flow through the die [24]. 

First, the drug and excipients are mixed in the same equipment used for the extrusion, or in a mixer. Possible excipients are bulking agents, matrix carriers, antioxidants, thermal lubricant, plasticizers and additives [25]. Subsequently, under heating, one or more components of the mix melt while the plastic mass is being extruded through the equipment (for example, ram extruder, single-screw or twin-screw extruder). As the extrudate is ejected from the machine, it cools and solidifies, and then is subjected to further downstream processes [1,26].

This continuous pharmaceutical process can be performed below the glass transition temperature (*T*_g_) of the mix [27], but generally is carried out above the melting temperature (*T*_m_) of the mix to reach the best operating procedures [28] and, in particular, the appropriate rheological properties [29].

The HME process offers many advantages and poses few disadvantages; these factors are summarized in Table 1.

During the design and optimization of an HME process, one must examine both the process factors (selection of the appropriate equipment, mass feed rate, process temperature, shear stresses, etc.), as well as the physicochemical factors (drug and excipient properties, possible interactions among all the components of the mix, mix physical state, and physicochemical stability). More recently, Process Analytical Technology (PAT) frequently combined with Design of Experiment (DoE) has proven interesting for successful process optimization, particularly for evaluating both process parameters and formulation factors. Design and optimization of an HME process can be described in terms of pre-formulation, formulation and process, and post formulation phases. 

In particular, this review will describe the pre-formulation, formulation and process, and post formulation factors that should be investigated while designing and optimizing a hot melt extrusion process (Table 2):

- The chemical and thermal stability of extrudates
- The solid physical state of extrudates
- The drug-polymer interaction
- The miscibility/solubility of the drug-polymer systems
- The rheological properties of extrudates
- The physicomechanical properties of films produced by hot melt extrusion
- The drug particle dissolution from extrudates

Before describing these factors in depth, a summary of the most widely applied technologies used in hot melt extrusion are considered. 

### 1.1. Hot Melt Extrusion: General Aspects

#### 1.1.1. Hot Melt Extrusion Technologies

Traditional extrusion processes have employed ram extrusion and screw extrusion technologies. More recently, injection molding and 3D printing technologies have been developed as natural evolutions of these classic HME technologies. 

Ram extrusion consists of a ram or piston that presses the feeding material, while moving inside a heated cylinder, generating high pressures [104]. 

In the more frequently used screw extruder system, the ram is substituted by a screw. Typically, it is composed of a conveying system for material transport and mixing, a die system, and downstream auxiliary equipment for cooling, cutting or collecting the finished product. The components within the extruder are the feed hopper, a temperature controlled barrel, a rotating screw, and systems for die heating and cooling [48]. 

Specifically, the barrel is composed of a feed zone, a compression zone, and a metering zone [26].

The pressure acting on the feeding mass increases while moving from the feed to the compression zone. Steady state flow is reached in the final zone: a constant flow is responsible for uniform thickness, shape and size of the extruded product [42].

Screw extrusion can generate higher shear stresses and more intense mixing than ram extrusion, thus affording higher homogeneity of the blend and higher process temperatures, which influence the final product characteristics [1]. 

There are single-screw and twin-screw extruders. The single-screw extruder operates in a continuous mode and is particularly suited for highly viscous materials, because it is able to generate very high pressures during extrusion. In the twin-screw extruder, two screws are generally arranged side by side, and each screw can rotate in the same or in the opposite direction [105].

The geometry of the twin-screw extruder favors a very high degree of mixing because the residence time of the mass is increased. The twin-extruder offers greater versatility, as it is able to process a wider range of pharmaceutical formulations. An important parameter of both single and twin-extruders is the length diameter ratio (L/D), which can affect the characteristics of the final product [42]. 

In a screw extruder, an electronic control unit makes it possible to set screw speed, process temperature, and pressure, in order to optimize the HME process. 

Co-extrusion is also possible when two or more materials are simultaneously melted in the same die [106]. 

Injection molding (IM) and 3D-printing (3DP) can exploit similar extrusion processes and thus these two technologies are frequently correlated to the more classic HM process. 

IM, when applied in the pharmaceutical industry, is a rapid and versatile manufacturing technique able to produce pharmaceutical dosage forms with different size and shape. The molten mass is injected under high pressure and temperature into a closed mold of a specific shape and size. The finished product cools down and/or solidifies inside the mold and is ejected at the end of the manufacturing cycle [107]. 

More recently, 3D printing, a layer-by-layer production of 3D objects from digital designs, has gained attention in pharmaceutical manufacturing with the first FDA approval of a 3D-printed drug product in August 2015 [108,109]. 

In particular, it is of interest for producing solid dosage forms containing high doses of drugs [110].

This technology may be particularly important for customized drug therapies (high dosage drug) by increasing patient compliance and adherence to the prescription [111]. 

The 3D printing can exploit different technologies, such as inkjet printing, extrusion, and electromagnetic radiation [112,113,114].

It can also be used in combination with HME. For example, a formulation containing immediate and sustained released caffeine was produced by these combined techniques in presence of methacrylic and cellulose-based polymers [35]. 

Five different shapes of pharmaceutical dosage forms containing paracetamol in presence of polyvinyl alcohol were obtained by a combination of HME and 3D printing. The different shapes influenced the drug release, with the pyramid shape affording the fastest release and the cylinder form yielding the worst [36]. 

Since in some cases the technology adopted during 3D printing is extrusion, the problems related to drug stability and drug interaction with excipients are the same. Thus, much of the information in this review is pertinent to both technologies. 

#### 1.1.2. Hot Melt Extrusion Scale-Up

HME is an easily scalable process, a feature that has contributed to the successful diffusion of this technology at the pharmaceutical industrial level. According to Dreiblatt [115], it is possible to apply different scale-up strategies for HME, including power scale-up, volumetric scale-up, and heat transfer scale-up. Each of these different methods are based on the consideration of specific parameters, such as mechanical energy, filling level and residence time, and heat transfer inside the barrel, respectively. 

In general, for continuous HME, scale up for pilot scale production can be achieved by: (a) running the process for a longer period; (b) increasing the material feed rate; (c) increasing the screw speed; (d) increasing the barrel size and screw diameter; (e) increasing the ratio of the outer and inner diameter of the screw; and (f) keeping similar screw elements on the extruder in the scale up from small to large scale [116]. 

The major drawback while scaling up in HME is that the shear forces can be modified, badly affecting the quality of the final product in term of physicochemical and thermal stability, drug dosage uniformity, and product uniformity in terms of particle size and distribution shape [43].

### 1.2. Pre-Formulation, Formulation and Process, Post-Formulation

Designing and optimizing an HME process takes into consideration pre-formulation, formulation and process, and post-formulation phases.

Pre-formulation studies that use such techniques as thermogravimetry, differential scanning calorimetry or hot stage microscopy have the advantage of saving materials, time and process costs, because only a small quantity of material is required and fast analytical methods are applied. It involves trying to expose the drug and/or the mix to experimental conditions similar to those reached during HME. One the main process parameters generally taken into account is the operating temperature. As it is not always possible to reproduce the real operating conditions, the pre-formulation phase must be followed by the real formulation and process phase.

The formulation phase and process optimization can take advantage of Process Analytical Technologies (PAT) and Design of Experiments (DoE), as in the quality by design (QbD) approach to optimize the HME process and develop successful formulations [28,117].

In particular, the continuous operation mode of HME allows for the application of PAT, in Compliance with Regulatory Affairs [50], with the possibility to easily validate, optimize, and scale up an industrial process.

On this point, Hitzer et al. [43] reviewed all the methods for equipping hot melt extruders for in-line, on-line, at-line and off-line measurements during HME. They considered all the analytical techniques able to provide fast and Near-infrared spectroscopy (NIR), Mid-infrared spectroscopy (MIR), Raman spectroscopy, UV-VIS spectroscopy, Terahertz spectroscopy, and finally ultrasonic spectroscopy. These techniques allow for monitoring and measuring process parameters such as temperature, feed rate, screw speed, pressure, as well as product properties such as drug concentration, crystallinity, homogeneity, and physical state.

QbD principles were applied to optimize the continuous production of solid lipid nanoparticles (SLN), through a combination of melt-emulsification and high pressure homogenization. A Plackett-Burmann screening design was adopted and the plan was built by selecting as variables drug concentration, lipid concentration, surfactant concentration, type of surfactant, type of lipid, screw speed, barrel temperature, and zone of liquid addition. An experimental plan was built selecting as dependent variables the average particle size, the polydispersity index, the zeta potential, and the entrapment efficiency. This approach made it possible to select the optimum conditions for the preparation of SLN with the best physicochemical and pharmaceutical properties and the minimum number of experiments [21]. 

In another study, QbD was applied to optimize the preparation of sustained-release formulations of paracetamol by HME. The experimental design was based on three independent variables (drug loading, screw speed, and feed rate), and three dependant variables (drug release after 6 h, drug release after 12 h, and particle size). The implementation of DoE (Design of Experiments) was possible by applying the PAT through in-line near-infrared (NIR) spectroscopy [37]. 

HME was efficiently applied in the production of tamper-resistant tablets, using a co-rotating screw extruder set in a standard configuration. Multivariate data analysis served to identify key critical process parameters such as feed rate, screw speed, and drug concentration, and define critical quality parameters of the tablet. The relationship between the critical parameters was established by means of DoE. NIR-spectroscopy was used for in-line monitoring of the material during the extrusion process [118].

The following chapters review several analytical methods and indicate the phase for which they are most appropriate. 

### 1.3. The Chemical and Thermal Stability of Extrudates

The high thermal and mechanical energy applied to the mix during extrusion can degrade drugs or excipients [118]. The WHO recommends that the chemical and thermal stability of the product be evaluated in order to identify any degradation species in medicinal finished products [51]; preliminary assessment should be conducted on the individual products and the mix during the pre-formulation phase. 

Chromatographic techniques such as high performance liquid chromatography (HPLC) are mainly devoted to evaluating the chemical stability of mix components subjected to high temperature and shear stresses. HPLC can be equipped with different detectors, the most important of which is mass spectrometry (MS), because it may enable identification of degradation products [56]. 

Given the high temperatures applied and the absence of solvents in the HME process, the most frequent unwanted reactions are oxidation and peroxidation, which affect drug chemical stability. HPLC is the most appropriate method for discovering any such drug degradation that may occur during the HME process [30,31,57]. 

In general, chromatographic techniques should be applied in all phases, then during pre-formulation, formulation, and post-formulation studies.

HPLC-MS was applied to evaluate the chemical stability of albendazole after treatment by spray drying or HME. Pre-formulation studies consisted in exposing albendazole under stressed conditions (acid, basic, oxidative, thermal environment). HPLC analysis results indicated that albendazole was stable under acidic conditions, but unstable under exposure to peroxides and strong alkali. Degradation pathways were also hypothesized. During formulation studies, albendazole was extruded by a twin-screw extruder at constant temperature of 120 °C, constant feed rate, variable screw speed (ranging between 100 and 300 rpm) and torque (from 5.4 to 14.2 Nm), and variable melt pressure (from 30 to 300 psi). The spray drying process was performed by dissolving albendazole in strong acids, at an inlet temperature of 100 °C and an outlet temperature of 60 °C. The authors found that under HME, but not under the spray drying process, the albendazole degraded, due to the higher temperature and shear stress applied [31]. 

Liu et al. [14] demonstrated the efficacy of HME for enhancing chemical stability of carbamazepine (CBZ)-nicotinamide (NIC) co-crystal solid dispersions. These were prepared with polymer carriers (polyvinyl pyrrolidone/vinyl alcohol PVP/VA, Soluplus^®^, or hydroxypropyl methyl cellulose HPMC) by HME (single screw extruder) at 160 °C and 30 rpm, and the chemical stability of the system was evaluated by HPLC. 

Another study used HPLC-MS to identify drug degradation caused when HME was used on gliclazide, a thermally labile drug, in the presence of the polymer Affinisol™ HPMC HME 100LV. Five HME runs in a co-rotating twin-screw extruder were performed, different for temperature, feeding rate, pressure, screw rotating speed, and torque. Stability studies carried out according to the Arrhenius equation on the different extruded batches indicated two hydrolysis degradation pathways of gliclazide, while the activation energy results indicated that gliclazide in the amorphous form had a much higher degradation rate than it did in the crystalline one. The authors were able to reduce the chemical degradation by changing and optimizing the process, in particular, by reducing the stress forces [58]. 

Meloxicam has been used as model poorly soluble drug, chemically instable at high temperatures. HME was performed on a twin-screw extruder and five different extrusion conditions were applied, by changing barrel temperature, feeding rate, and screw speed. The drug chemical stability was increased by melting point suppression and adjustment of microenvironmental pH, in addition to process parameter optimization. HPLC-MS identified two degradation pathways of meloxicam due to hydrolysis reactions when the drug extrusion was performed in presence of Kollidon^®^ VA64. The authors found that melting point suppression did not prevent meloxicam degradation; instead, they were able to increase the drug chemical stability through process parameter optimization, specifically, by establishing a minimum processing temperature (110 °C), and providing a basic pH environment during extrusion [59]. 

HPLC photodiode array (HPLC-PDA) and gas chromatography coupled mass spectrometry (GC-MS) methods proved effective in identifying degradation products of hydroxypropyl methylcellulose phthalate (HPMCP) caused by HME (co-rotating twin-screw extruder). Eight batches were processed, varying barrel temperatures from 160 to 190 °C and two screw speeds (80 or 100 rpm). Several impurities were identified and a degradation mechanism for HPMCP was proposed [60]. In particular, acetic, succinic, phtalic acids, and phtalic anhydride were identified, and in particular two environmental analytical impurities were recorded: dimethyl phthalate and methyl benzoate. 

In another such study, HPLC-PDA was used to determine the chemical degradation of naproxen mixed with PVP K25 after HME (co-rotating twin-screw extruder, barrel temperature 90 or 120 °C, screw speed 150 rpm) or spray drying (inlet temperature 100 °C, outlet temperature 60 °C). Minimal degradation (0.80 ± 0.01%) was observed subsequent to HME, while none at all was observed in association with the spray drying process [61]. 

In addition to HPLC, gel permeation chromatography (GPC) has also been used to evaluate the chemical stability of substances processed under HME. GPC is a type of size exclusion chromatography (SEC) that separates analytes on the basis of size. It is particularly important for the characterization of polymers, the most important parameters of which are the number average molecular weight and the dispersity index. Changes in these parameters during processing could be due to polymer degradation. During HME, polymers are subjected to both thermal and shearing stresses, and in particular high temperatures may favour the depolymerization of polymer chains, while shearing effects of the screw could promote polymer chain breakage.

An example of GPC used to evaluate chemical stability of substances processed under HME is the work on sustained release pellets of diltiazem hydrochloride formulated in presence of different polymers such as ethylcellulose, cellulose acetate butyrate, poly(ethylene-co-vinyl acetate), and polymethacrylate derivative [18]. Assessment of the polymer chemical degradation before HME and during HME was conducted using GPC. The authors found that polymer molecular weight and polydispersity indices remained lower and constant under extrusion, confirming that only slight degradation occurs during extrusion.

In another study, GPC was applied to evaluate the stability of polymer films containing hydroxypropylcellulose and polyethylene oxide that were extruded with or without vitamin E TPGS (d-α-tocopheryl polyethylene glycol 1000 succinate) as an absorption enhancer for possible controlled release formulations [62]. In this case, results confirmed that vitamin E TPGS decreased the degradation and chain breakage of the polymer blend and helped prevent thermal oxidation of polyethylene oxide. 

Another example of the use of GPC was provided by a study to formulate sustained release chlorpheniramine maleate (CPM) tablets by a HME process on polyethylene oxide (PEO), a free flowing and thermoplastic homopolymer, generally used to prepare such sustained release dosage forms. GPC was used to evaluate the thermal stability of PEO under different storage temperatures and under extrusion, by measuring PEO average molecular weight. PEO was firstly stored at 40, 60, and 80 °C for 15 days. Degradation was accelerated at the higher storage temperatures, and was particularly evident over the polymer melting temperature (80 °C), when both crystalline and amorphous phases liquefied and oxidative degradation accelerated. HME was then performed on a single screw extruder, the screw speed varying from 10 to 60 rpm. Under HME, PEO was sensitive to both process temperature and screw speed, and the chemical degradation was both thermal and mechanical. Vitamin E could protect PEO from oxidation when it is molecularly dispersed in the polymer [4].

Guo et al. [44] selected diflunisal as a heat-sensitive model drug to verify whether the formation of hydrogen bonds between the drug and the polymer minimized the chemical degradation of solid dispersions prepared by HME. Diflunisal was mixed with different polymeric carriers (PVP K30, PVP VA64, hydroxypropyl methylcellulose and Soluplus^®^) and subjected to HME (single screw extruder, operating temperature 160 °C, screw speed 150 rpm). During post-formulation studies performed by placing the extruded material at 40 °C, 75% RH for three months, the authors demonstrated by HPLC that the percentage of impurities in mixes of diflunisal with any polymers was lower than 0.3%, indicating that thermal degradation was effectively minimized. 

In many cases, the thermal stability of a mix is preliminary evaluated through thermal techniques, such as thermogravimetry (TGA) and differential scanning calorimetry (DSC), which can be applied alone, or together with the previously described methods. TGA is particularly interesting because it can be used to follow changes in the weight during heating, which can be due to molecule degradation (frequently before, during, or after a melting event), corresponding to a loss in molecules formed during chemical degradation, such as water, carbonic acid, amines, or smaller fragments of the original molecule. TGA has the advantage of being a very rapid and predictive way to identify possible thermal degradation during pre-formulation studies. While the previous techniques are generally applied after the HME has actually been carried out, serving to confirm that the mix has remained stable during the process, TGA is frequently applied during the design of the process.

Besides, one of the most important variables during HME is the process temperature, which depends on several factors, such as the melting of the mix and the “fluidity” of the mass to be extruded. Frequently, the process design requires that the operating temperature be increased in order to obtain the rheological properties needed to allow the extrusion. Even if this temperature may be decreased by the addition of specific additives such as plasticizers, nevertheless relative high temperatures are often necessary, and they affect the thermal stability of the mix. Thus, during the process design, a balance between process feasibility and possible mix degradation must be considered. TGA makes it possible to predict thermal stability and identify the temperature limit necessary in order to avoid thermal degradation. 

For example, TGA, used to determine the thermal stability of diflunisal [44], revealed serious decomposition and weight loss over the melting temperature of 215 °C. This result was important in demonstrating that diflunisal should not be subjected to HME at such a high temperature; it also showed that amorphous solid dispersions were successfully obtained at an extrusion temperature of 160 °C, 55 °C lower than the diflunisal melting temperature.

TGA was also used to assess the thermal stability of polyethylene oxide (PEO) in sustained release tablets prepared by HME [4]. The authors were able to establish that depolimerization by oxidation of PEO started at nearly 200 °C with the formation of smaller polymer segments. They also observed that the lower the polymer molecular weight, the faster the degradation rate. 

In another study, TGA was used to follow the thermal degradation of gliclazite as pure drug or in presence of the polymer Affinisol™ HPMC HME 100LV. The pure drug started to degrade over 165 °C, which corresponds to the melting temperature recorded by DSC. The weight loss from 165 to 240 °C was less than 10%, but HPLC confirmed that drug degradation indeed took place. This information was key for optimizing the HME process. The authors were able to use a lower processing temperature, far below the degradation temperature, based on their findings that the gliclazite and Affinisol™ exhibit good miscibility, and that the solubilization of the drug in this polymer serves as a plasticizer [58]. 

TGA was also used to determine the degradation temperature of several polymers (Soluplus^®^, Kollidon^®^ VA64, Kollidon^®^ 12 PF, and Affinisol^®^ HPMC), which would have the ability to form amorphous solid dispersions with the drug [45]. 

Several strategies have been considered to overcome chemical degradation of temperature sensitive drugs during extrusion. Above all, intermolecular interactions between drug and polymer create new conditions that require lower operating temperatures, and consequently depress the drug melting temperature or lower polymer viscosity [52].

To counteract this phenomenon, one strategy is the use of a polymer that suppresses the melting point of the drug substance by establishing hydrogen bonds between the drug and the polymer, thus enabling the drug to dissolve into the polymer at a temperature below its melting point [14,44].

Another approach is to use plasticizers or surfactants during extrusion to reduce the glass transition temperature of the polymer [32,63]. In some cases, the drug itself can act as a plasticizer [64].

Another strategy is the injection of a supercritical fluid into the extruder barrel [59], because it acts as polymer plasticizer, decreasing polymer viscosity, melting temperature, and glass transition temperature [49].

### 1.4. The Solid Physical State of Extrudates

Key to the characterization of hot melt extrudates is the evaluation of their solid physical state (amorphous, crystalline or partially amorphous), because this in turn influences stability during the product shelf life, crystallization tendency, drug dissolution, and drug bioavailability. Evaluation of the solid physical state is extremely important in relation to HME, since this process frequently promotes the disruption of the crystal lattice and the recovery of an amorphous or partially amorphous solid.

Actually, in an HME process, the drug-polymer system can be considered as the dispersion of a drug into a polymeric matrix where the drug can be present as non-dissolved particles, as solid solution, or as a combination of both [45,46,47].

To follow modifications at the solid state of a drug–polymer system under the HME process and also to evaluate the physicochemical stability of the system during its shelf life, various techniques are of prime importance, among them hot stage microscopy (HSM) or hot stage polarized light microscopy (HS-PLM), atomic force microscopy (AFM), X-ray powder diffraction (XRPD), differential scanning calorimetry (DSC), and modulated temperature DSC (MTDSC).

HSM combines information from microscopy and thermal analysis, making it possible to follow the behaviour of a solid material as a function of temperature and time. Thus, researchers can obtain information about particle size and particle morphology, while also learning about melting or crystallization or more in general solid transformations during heating.

In addition to the benefits offered by HSM, HS-PLM also offers the advantage of a polarized light that better highlights transformation of the material. Both techniques are interesting because of their speed, and thus could be used preliminarily to predict the physical state of the mix under heating, specifically the dissolution of the drug into the polymer, solid state transitions, and melting. 

AFM is a technique that exploits a scanning probe capable of locally detecting properties of a material, such as solid-solid separation, and is particularly interesting for investigating surfaces on an atomic scale [65]. 

Another technique of prime importance for the characterization of a solid material is X-ray powder diffraction, which shows diffraction peaks whose sizes vary according to the state of the crystal lattice of the solid or a halo corresponding to a completely amorphous solid. When a solid exists as partially crystalline, smaller diffraction peaks than those corresponding to the 100% crystalline solid are present at the same distances. Amorphous or partially amorphous solids tend to crystallize, and, interestingly, XRPD also allows crystallinity quantitation. Several methods using XRPD have been developed to accurately determine the degree of crystallinity of a material [54], among them Ruland’s [66], and Hermans and Weidinger’s [67] methods. 

In particular, XRPD makes it possible to distinguish solid solutions, in which the drug is amorphous, and solid dispersions, in which the drug should also be present in a partially crystalline form [1].

Differential scanning calorimetry (DSC) is another technique widely applied for the characterization of the solid state (crystalline and amorphous) of a mix subjected to HME, as it provides information about the melting temperature (*T*_m_), the glass transition temperature (*T*_g_), the crystallinity degree or the minimal amount of amorphous form in crystalline solids. DSC is able to determine the degree of crystallinity of a drug, classically with a detection limit of 1%.

In DSC, a linear or isothermal temperature program is applied as a function of time or temperature simultaneously to a sample and an empty pan (the reference), making it possible to measure the temperature and the heat flow associated with state transitions that occur during a specific temperature program in the sample [54].

Modulated temperature DSC (MTDSC) [72], an evolution of DSC, applies to a sample a sinusoidal (modulated) heating signal on a linear scan (or isothermal) program, thus allowing the separation of the total heat flow response into its reversing and non-reversing components. 

In practice, MTDSC can highlight phenomena that otherwise could be masked during heating, for example a glass transition masked by a desolvation endotherm [54]. 

In general, these techniques are never used alone, but conclusions about solid transitions are better confirmed by the concomitant use of more than one technique.

The literature provides many examples of the use of these techniques to follow modifications at the solid state of a drug-polymer system under the HME process. 

In one pre-formulation study, for example, hot stage polar microscopy was used to track the dissolution of diflunisal in different molten polymers (PVP K30, PVP VA64, hydroxypropyl methyl cellulose, and Soluplus^®^) at 160 °C, a temperature far lower than that at which the pure drug melts (215 °C) [44]. With this information, the HME experimental temperature could be set to a lower range, thus preventing possible drug degradation. 

In another pre-formulation study, HS-PLM revealed traces of crystalline meloxicam suspended in a glassy PVP/VA polymer, though X-ray beams could not sufficiently penetrate the sample to detect the crystals [59].

HS-PLM was also used to monitor the behaviour under extrusion of theophylline in a polymer mix. At 200 °C, the polymer mix was completely melted, but tiny crystals of theophylline were still present, as the melting temperature of theophylline is 270–275 °C. In extrudates, theophylline crystals appeared smaller and narrower in size distribution compared to crystals of physical mixture, because extrusion acted as mechanical milling [38]. 

In yet another pre-formulation study, HSM and DSC were used together to confirm that carbamazepine and nicotinamide could form co-crystals in situ in a polymer matrix during the heating process. Co-crystals melted at 160 °C, which was lower than the drug melting temperature (approximately 190 °C) and, once the melt was cooled, co-crystals were completely dispersed in the polymer as amorphous solid dispersion. Since TGA demonstrated the thermal stability of the mixture under the operating temperature, the authors proposed that all the components of the mixture should be melted at 160 °C to favour the formation of an amorphous solid dispersion [14]. 

In a recent work, AFR and MDSC were used to select the best operating conditions for obtaining a single-phase amorphous material, by extruding d-*α*-tocopherol polyethylene glycol 1000 succinate (TPGS 1000) with copovidone in a co-rotating twin-screw extruder. Several process parameters such as screw speed, cooling conditions, and composition were correlated to the solid physical state of the mix [73]. At 10% *w*/*w* of TPGS 1000 in copovidone and a screw speed of 600 rpm, a single phase was observed, while when the screw speed was decreased to 300 rpm, a separation phase was evident, because of insufficient mixing. Reducing the TPGS 1000 to a 5% *w*/*w* produced a single phase product even at lower screw speeds (300 and 150 rpm).

In an earlier study, XRPD, DSC and MTDSC were successfully used to evaluate the solid state of extrudates of paracetamol with Eudragit^®^ EPO or Kollidon^®^ VA64 at different proportions, chosen to mask the unpleasant taste of the drug. These techniques served to determine the amorphous or crystalline character of the solid materials, calculate the degree of crystallinity, and measure the T_g_. The components were firstly mixed in a Turbula^®^ mixer and then extruded in a single screw extruder; the screw speed was constant at 15 rpm and barrel temperature varied between 100 and 115 °C. This study revealed that the degree of crystallinity of paracetamol mainly depends on the polymer used in HME, and concluded that the pleasant taste was directly correlated to the solid state of the paracetamol. The best taste was that with VA64 [68].

In yet another study, XRPD was used to evaluate the crystallinity degree of ketoprofen mixed with sulfobutyl ether *β*-cyclodextrin; the results indicated that the mix was completely amorphous when treated under HME at a temperature very close to the ketoprofen melting temperature [69].

XRPD was also exploited to assess crystallinity quantitation in a mix of naproxen-povidone treated under HME, in this case through the Ruland’s method, with the T_g_ calculated by MTDSC. Haser et al. [61] formulated naproxen in presence of povidone K25 at different percentages under HME or spray drying. By XRPD, the authors proved that spray dried mixes were more susceptible to re-crystallization. XRPD also served to detect the crystalline percentage in the two systems and follow crystallization during storage. 

On the contrary, spray drying was the preferred preparation method for recovering drug-amino acid mixtures in an amorphous state. In particular, Lenz and co-workers [119] formulated an indomethacin-arginine mix in presence or not of povidone as polymer. By XRPD they confirmed that arginine is a good stabilizer for amorphous indomethacin [70], and observed that the polymer further increased the mix stability during storage. 

In another study, a poorly water-soluble drug (non-referred name) was formulated as an amorphous solid by solvent evaporation and then melt-extruded in presence of PVP K30, a polymer able to prevent drug crystallization. The melt extrusion was produced in a small-scale twin extruder at temperatures between 178 and 184 °C. DSC was used to evaluate the thermal behaviour and measure the glass transition temperature (*T*_g_) of the mix. First of all, it was possible to verify that the drug acted as a plasticizer for the polymer, lowering the *T*_g_ of the mix. Subsequently, the stability of the drug in its amorphous state was proven at different drug-polymer ratios, and an increase in bioavailability of the drug was proven in vitro and in vivo [64].

In a different work, DSC was not effective in detecting the physical state of theophylline formulated in a polymer mix, because the carrier degraded at 220 °C, below the drug melting temperature (270–275 °C). Thus degradation curves masked the drug melting temperature. Nonetheless, XRPD made it possible to highlight the presence of typical peaks for crystalline theophylline, confirming that theophylline did not melt or solubilise in the polymer during the HME [38].

XRPD was used in a study to characterize the solid dispersions of diflunisal with different polymers, showing them to be fully amorphous. In contrast, physical mixtures with the same proportions of diflunisal and polymers still had typical diffraction peaks of the drug [44]. 

Mitra et al. [71] demonstrated under HME the formation of amorphous solid dispersions between a poorly soluble model drug A and several polymers, among them the copolymer copovidone or different grades of the polymer hydroxypropyl methyl cellulose acetate succinate, specifically HPMCAS-HF and HPMCAS-LF. XRPD and DSC proved the formation of amorphous solid solution with the disappearance of peaks typical of the drug. MTDSC permitted the calculation of the *T*_g_ of the mixes, which was higher than that of pure drug in its amorphous state. Study of physical stability under stressed storage conditions highlighted that an amorphous-amorphous drug-povidone separation is a precursor sign of drug crystallization. 

### 1.5. The Drug-Polymer Interaction

Limitations in HME are drug thermal degradation, limited physical stability of the mix, and precipitation of the drug during dissolution. These limitations can be circumvented when drug and polymer establish important or stable interactions, such as ionic interactions, hydrogen bonds, dipole-dipole, and Van der Waals interactions [52]. 

In the previous section, several techniques for evaluating the solid physical state of drug–polymer mixes were described. However, these techniques are only able to provide information on the physical state of the material under processing (solid, liquid, amorphous, partially amorphous), without explaining the reasons for the physical state or for transitions from one state to another. To do so, it is necessary to explore in depth the possible interactions between the drug and the polymers, or in general the mix components.

Among the various methods for elucidating the interaction mechanism at a molecular level, spectroscopic techniques are the mostly widely used. 

A pre-formulation study used FTIR spectroscopy to determine the distribution of theophylline at the microscale level in a polymer mix extrudate. Taking into account the wave number of 1659 cm^−1^, the only one visible for theophylline in the formulation, it was possible to conclude that the drug was distributed homogeneously in the polymer matrix, and thus predict that the drug would be released homogeneously from individual pellets [38]. 

Fourier-transform infrared (FTIR) spectroscopy can directly monitor the vibrations of the functional groups that characterize molecular structure and monitor the progress of chemical reactions.

For example, in one study, the solid state drug polymer interactions between indomethacin, itraconazole, or griseofulvine and methacrylated polymers subjected to HME in laboratory scale equipment were identified by FTIR analysis. The supersaturation was confirmed by the stretching of the carbonyl peaks at higher values, and thus the authors concluded that the drug dissolution rate was determined by the drug-polymer interaction, which favoured the formation of an amorphous solid dispersion and particle dissolution [74]. 

In another study, FTIR spectroscopy highlighted the shifts of C=O and C–OH stretching vibrations for diflunisal with PVP K30 and diflunisal with PVP VA64 HME products, which indicated the formation of hydrogen bonds between the drug and the polymer. Given this strong interaction, it was possible to lower the process temperature, thus increasing the chemical stability of the mix [44].

FTIR analysis was also used by Salmoria et al. [10] to confirm interaction between ibuprofen and polycaprolactone (PCL) during HME; the technique identified a broad band from 3300 to 3100 cm^−1^, corresponding to the O–H bending. This interaction could explain the decrease in crystallinity degree calculated on the basis of information from DSC and XRPD studies. 

Most recently, attenuated total reflectance-Fourier transform infrared (ATR-FTIR) spectroscopy was used to evaluate the molecular interaction between naproxen and PVP K25 [61]. The hydrogen bond formation between naproxen and the polymer was proven by the fact that the band at 1683 cm^−1^ from crystalline naproxen shifted down to 1674 cm^−1^. 

Finally, in an interesting study, X-ray photon spectroscopy (XPS) was applied to investigate the interactions between cationic drugs, propranololor diphenhydramine hydrochlorides, and anionic methacrylic acid-base methyl methacrylate copolymers, processed under the HME technique. Molecular modelling predicted the existence of two possible H-bonding types, which were then confirmed by XPS. Differences in the intensity of the carbon peak suggested a reaction of ester/carboxylic acid functionalities from polymers and drugs to yield C–O or C–O–C like hydrocarbon chains in the extruded materials. The magnitude of the intermolecular interactions varied according to the drug–polymer miscibility [75].

As indicated in Table 1, one of the advantages of HME is its continuous process mode. In line with the FDA recommendation to continuously monitor the process, appropriate techniques must be used, for example near infrared (NIR) spectroscopy, which can be appropriate for process analytical technology (PAT) [50].

NIR was confirmed as a potent tool in PAT monitoring by Islam et al. [76], who used NIR spectroscopy to conduct in-line monitoring of the extrusion of different percentages of indomethacin in presence of Soluplus (polyvinyl caprolactam-polyvinyl acetate-polyvinyl-glycol graft copolymer) and Kollidon Va64 (vinylpyrrolidone-vinyl acetate copolymer). The drug was converted into its amorphous state during processing, through the formation of a hydrogen bond between the drug and polymer. The authors suggested that techniques such as DSC and FTIR could be helpful in confirming the NIR results, but due to the formulation instability, only Raman spectroscopy proved effective [77]. 

Thus, Raman spectroscopy is another potent tool for explaining the solid-state of a mix while processing. In addition, it has proven to be an effective PAT-tool for solid-state monitoring during processing. Van Renterghem et al. [78] put this tool to good use in their recent study of high content metoprolol tartrate formulation. Processing above the drug melting temperature in presence of Eudragit^®^ RS PO produced an amorphous mix, whereas processing below the drug melting point lead to solid dispersions with partially amorphous/crystalline drug. The authors explained that when a greater quantity of the drug was dissolved in the molten polymer, the Raman peak intensity of the COOH vibration band decreased by simultaneously shifting, indicating O–H interactions between the drug and the polymer. 

They then included in-line Raman spectroscopy in the HME apparatus, in order to continuously monitor the process and investigate how its parameters influenced solid-state transformation of a crystalline drug during HME into an amorphous polymer. It was concluded that a high drug load processed at temperatures over the drug melting point allowed for the recovery of a completely amorphous product, while processing below drug melting temperature lead to solid dispersions in which the drug was partially crystalline. 

Nuclear magnetic resonance (NMR) was used to determine the interaction between naproxen and PVP K25 subjected to HME or spray drying. The 30% naproxen amorphous solid dispersions obtained by HME showed good miscibility with the polymer, while the 60% naproxen spray dried sample showed high phase separation, expressed as a great difference in spin-lattice relaxation times for naproxen and polymer, which can be explained by the crystalline content of the spray dried material [61].

### 1.6. The Miscibility/Solubility of Drug-Polymer Systems

The miscibility of drug and excipients, which is typically explored during pre-formulation studies, is essential to guarantee their interaction, as shown in the previous examples. It also has positive effects on process parameters and dissolution behaviour, as will be explained later. 

The drug/excipient miscibility can be estimated by calculating the solubility parameter (δ), which is the most common approach for quantifying the cohesive energy of a material [79,80,81], that is, the amount of energy necessary to separate the atom of a solid or liquid to a distance where no interactions can occur between atoms.

Several methods can be applied to calculate solubility parameters, for example, the Hansen method. In one pre-formulation study, the Hansen solubility parameters for meloxicam and Kollidon^®^ VA64 were calculated, based on molecular structure and melting points, using a specific software. The results indicated differences in the solubility parameters for meloxicam and Kollidon^®^ VA64; this information made it possible to predict miscibility and melting point depression [59].

It must be noted that the formation of amorphous solid dispersions (ASD) can be reached by solubilizing the drug in the polymer rather than necessarily proceeding through a drug melting process [58]. In this case, the miscibility between the drug and the polymer is crucial.

Two other approaches for calculating solubility parameters were demonstrated in a study by Desai et al. [82,83], on binary and ternary mixtures of indomethacin, Eudragit^®^ E PO with different plasticizers (stearic acid, glyceryl behenate, or polyethylene glycol 8000). They calculated the solubility parameters by using the Van Krevelen and Fedors [84] methods. Their results proved that all the binary (polymer-plasticizer, or drug-plasticizer) and ternary systems (polymer-drug-plasticizer) were completely miscible, making it possible to predict the formation of a one-phase system. These results were also confirmed by thermal analysis. In this case, DSC was used to compare experimental and theoretical *T*_g_ of the mixtures. The theoretical value was determined by the Gordon-Taylor equation and was based on the volume additivity of solids. Correspondence between predicted and calculated values confirmed drug–polymer miscibility, while deviations suggested immiscible mixes.

Molecular modelling software was used to calculate solubility parameters of drugs (indomethacin, itraconazole, or griseofulvin) in different methacrylic polymers. The results made it possible to predict that all the drugs would be miscible with the selected polymers, except itraconazole with Eudragit^®^ E PO [74]. In fact, DSC later confirmed the limited interaction between the latter two, revealing double glass transitions for them, thus confirming the predictions of the modelling.

In a study uniting thermal analysis with a combination of the Hoy and Hoftzyer/Van Krevelen methods for Hansen solubility parameter calculation, Forster et al. [55] estimated drug/excipient miscibility of indomethacin and lacidipine with polymeric and non-polymeric excipients. Miscibility was experimentally determined by DSC and HSM. Differences in the drug/excipient solubility parameters lower than 7.0 MPa^1/2^ indicated significant miscibility and the formation an amorphous solid solution, while differences higher than 10 MPa^1/2^ indicated a lack in miscibility and dispersion of the amorphous drug in the crystalline excipient. Their experimental results were in agreement with their solubility parameter predictions. 

In a more recent study, solubility parameters were the starting point for confirming the miscibility of a model system composed of a binary mix of indomethacin and Eudragit^®^ E PO. The efficiency of several plasticizers in the HME process was evaluated, specifically stearic acid, glyceryl behenate, and polyethylene glycol 8000 [82]. 

In another study, sorbitol, a water-soluble plasticizer, was selected to modify the thermal properties during HME of polyvinyl alcohols of different degrees of hydroxylation. The complete melting of the polymer-plasticizer binary system was necessary to promote the molecular interactions and this influenced the HME process: the formation of a one phase solid dispersion made it possible to decrease the extrusion temperature from 180 °C, necessary to promote the complete melting of the physical mix, to 140 °C, thus imparting greater thermal stability to the extrudate [39].

### 1.7. The Rheological Properties of Extrudates

In general, high process temperatures are necessary during HME to soften the polymer and favour the diffusion of the drug molecules through the polymer chains [52]. The dissolution of the drug molecules into the polymer is important for obtaining an amorphous solid dispersion and an increased drug dissolution rate.

The evaluation of viscoelastic properties of a material to be subjected to HME during pre-formulation studies is essential for predicting the behaviour of the molten material under extrusion and may help formulators in determining whether a mixture can be processed under HME.

An example of such an evaluation is the study by Meena et al. [85] in which oscillatory rheometry was used to assess the viscoelastic properties of cellulosic ester polymers with varied degrees of substitution and different substituent groups. The combination of rheometry with thermal methods during the pre-formulation study made it possible to predict the behaviour under HME. The authors observed that both glass transition temperature and viscoelasticity depend on chain length, molecular weight, and degree of substitution on the main polymer chain. They also found that an increase in chain length or in molecular weight increased *T*_g_ and viscosity. Most of the cellulose polymers were characterized by high viscosity between *T*_g_ and the degradation temperature, and thus could not be extruded. The authors concluded that HME can be used with these systems only if an appropriate plasticizer is added.

Another example is a study by Chokshi et al. [29] to evaluate the viscoelastic properties of the model drug indomethacin and several polymers (Eudragit EPO, PVP/VA, PVP K30, and poloxamer 188), in order to assess the suitability of a HME process. The study started by assessing the drug–polymer miscibility. DSC was used to measure the *T*_g_ of the binary mixes, and measured values were compared to those predicted through the Gordon-Taylor equation. Results showed only one *T*_g_ for all the tested binary mix ratios of indomethacin with EPO, PVP/VA and PVP K30, and two *T*_gs_ for indomethacin with P188, leading the authors to conclude that the EPO, PVP-VA and PVP K30 binary mixes were completely miscible, while P188 was partially miscible. The rheological study was then performed using a torque rheometer at different shear rates and temperatures. For the EPO, PVP/VA and PVP K30 binary mixes, the zero rate viscosity was lower than that of the pure polymer, because the miscible drug disrupts the polymer network. On the contrary, indomethacin with P188 exhibited higher zero rate viscosity because the drug is only partially miscible with the polymer. On the basis of the Gordon-Taylor equation, the authors suggested that the indomethacin-EPO mix exhibits antiplasticization behavior, while indomethacin with PVP/VA and PVP K30 exhibits a plasticization effect. These results were then confirmed by rheology studies focused on the activation energy value: the decrease in activation energy for indomethacin-PVP/VA with increase in drug concentration confirmed the plasticizing effect, while an increase in activation energy with drug concentration for indomethacin-P188 confirmed the presence of a two phases system and thus partial miscibility. On the basis of these results, the authors were able to predict the feasibility of HME and the best conditions for performing the process, in terms of selected polymer, drug-polymer ratio, and such operating conditions as temperature.

Viscosity was a key parameter examined in a study by Wu and McGinity [63]. During HME of Eudragit RS PO in presence of methyl paraben, they observed that when they increased the amount of methyl paraben, melt viscosity decreased. They attributed this to its plasticizing effect, which was also proven by the decrease in the *T*_g_ of the polymer in presence of methyl paraben, which increased the mobility of the polymer chain. They explained that the plasticizing effect is due to the interaction between the hydroxyl group of the methyl paraben and the ester group of the polymer, as proven by the chemical shift observed by solid-state NMR.

In another study, torque rheometry was used to measure the viscosity of the melt of mixes of a poorly soluble drug with different hydrophilic polymers prepared in presence or in absence of surfactants (Tween 80, docusate sodium, Pluronic^®^ F68, sodium lauryl sulfate). The authors observed that viscosity decreased due to the plasticizing effect of the surfactants on the polymer. Thermal analysis proved that surfactants caused salvation/plasticization; specifically, they showed a reduction of the drug melting temperature and a reduction in the *T*_g_ of the mixes [32].

In another study, Indomethacin (IND), Itraconazole (ITZ), and Griseofulvin (GSF) were mixed with one of the following hydrophilic polymers: Eudragit EPO, Eudragit L-100-55, Eudragit L-100, HPMCAS-LF, HPMCAS-MF, Pharmacoat 603, and Kollidon VA-64. Among the other characterizations, rheological analysis of drug–polymer physical mixtures (PMs) was performed using cone-plate geometry to assess the lowest temperatures at which it was possible to soften the samples and perform the rheological analysis. The zero rate viscosity (η_0_) was dependent on the melting temperature and consequently the state of the drug in the polymer at the softening temperature. The η_0_ of physical mixtures was useful to estimate the processing conditions for HME and to produce transparent glassy HMEs from most of the physical mixtures. The rheological results were helpful to optimize the HME process, by selecting the speed and temperature of extrusion process [74]. 

Rheological and thermal characteristics of mixtures containing antiretroviral drugs (zidovidine or lamivudine) with ethyl cellulose in presence of different plasticizers (triethylcitrate—TEC or polyethylene glycol—PEG-6000) were evaluated to assess the feasibility of the HME process. Physical mixtures were characterized by DSC and parallel plate oscillatory rheometry. The thermal and rheological analyses made it possible to evaluate the plasticizing effect of drugs and plasticizers on mixtures with ethyl cellulose and their appropriate proportions in the formula, and thus to define the conditions for a successful HME process [86].

Rheological studies of polyvinylpyrrolidone-based polymers and copolymers that can be used in HME were performed at different temperatures to calculate storage modulus (G′), loss modulus (G″), tan δ and complex viscosity (η). The results may help in the selection of polyvinylpyrrolidone-based polymers for HME. In particular, the tan δ = 1 values indicated that the polymers converted from solid to liquid forms with increasing temperature, while the viscosity values decreased with increasing temperature. These results made it possible to select to most appropriate temperature for HME [53].

### 1.8. Physicomechanical Properties of Films Produced by Hot Melt Extrusion

Elastic modulus, tensile strength and elongation of extruded products are other important parameters to be evaluated because they play a vital role in product processability, drug product stability and drug release. These parameters can be differently affected by the formulation, that is, the presence of plasticizers, the drug loaded, and the process used to produce the film. Several examples can be considered.

A single screw extruder was used to prepare films of hydroxypropylcellulose (HPC) films containing hydrocortisone 1% or chlorpheniramine maleate 1% as drugs, or plasticizers (polyethylene glycol 8000—PEG8000 2%, triethyl citrate—TEC 2%, acetyltributyl citrate—ATBC 2%, and polyethylene glycol 400—PEG 400 1%). The thermal properties of the films were evaluated by DSC, which made it possible to measure the *T*g, while physicomechanical properties of the films were evaluated for tensile strength (TS), percentage elongation (%E), and Young’s modulus (YM). Those characterizations were helpful for evaluating the stability of the films, showing changes in *T*g and physicomechanical properties after 6 months [87].

Another study determined the physicomechanical properties (tensile strength and percent elongation) of films containing hydroxypropylcellulose (HPC) and polyethylene oxide (PEO) with and without Vitamin E TPGS as an additive. The study showed that tensile strength decreased with increasing concentrations of TPGS, and the %E increased over 3-fold when compared with the HPC/PEO film that contained no additives. The Vitamin E TPGS facilitated the processing of the HPC/PEO films by decreasing the barrel pressure, drive amps, and torque of the extruder equipment [62]. 

Another work aimed to evaluate the possibility of using maltodextrins (MDX) with a low dextrose equivalent as film forming material for oral fast-dissolving films. Glycerin was used as plasticizer able to form flexible films, while cellulose microcrystalline and piroxicam loaded as model drug decreased the film ductility. Nevertheless, films kept good flexibility and resistance to elongation and a high loading capacity showing satisfactory drug dissolution rates [88].

Polyethylene oxide (PEO) and model drugs guaifenesin (GFN) or ketoprofen (KTP) were mixed in a V shaped mixer and then extruded in a single screw extruder at constant speed and varied temperature to obtain films of 0.3–0.45 mm. The mechanical properties of the films, such as the percent elongation and tensile strength, were determined in a tensile strengthener and the study revealed that percent elongation decreased with increasing GFN concentrations and significantly increased with increasing levels of KTP. Both GFN and KTP decreased the tensile strength of the extruded film [89].

In formulating an orodispersible film (ODF), it is important for polymer choice to strike a balance between mechanical properties and release rates. 

Hydroxypropyl cellulose (HPC) films were prepared in presence of solubilising polymers (Kollidon^®^ VA 64 or Soluplus^®^) in a co-rotating twin screw extruder rotating at 100 rpm and at a temperature lower than 140 °C to avoid the degradation of the components. Two different drugs, indomethacin and chlorpheniramine, were loaded. Experiments were designed by a 2^3^ full factorial plan, where three independent factors were considered: the solubilising polymer ratio and type, and the model drug. The mechanical properties of the films were studied in terms of tensile strength, elongation percentage, and Young’s Modulus. The mechanical properties were influenced by both the drug and solubilising polymer. Both drugs exhibited plasticising effects and had higher film ductility and lower film stiffness. Kollidon^®^ VA 64-containing films performed better in terms of drug release, whereas Soluplus^®^-containing films had better mechanical properties. The drug dissolution rate was improved by decreasing film thickness [33].

### 1.9. The Drug Particle Dissolution from Extrudates

One of the most important advantages of HME is the possibility to enhance the solubility of poorly soluble drugs and thus increase their dissolution rate. In an amorphous solid dispersion, the drug in its amorphous state exists in a high-energy state [90], allowing for higher solubility and thus higher dissolution rate, according to the Noyes-Whitney equation [91]. 

Drug particle dissolution in extrudates was the focus of a study on valsartan, a poorly soluble drug, extruded in presence of Soluplus^®^ and d-*α*-tocopherol polyethylene glycol 1000 succinate in different conditions to obtain solid dispersions with the best oral bioavailability [34]. In vitro release profiles of valsartan, a compound that contains a carboxylic acid function and thus with a pH dependent solubility, were determined at different pHs (1.2, 4.0, and 6.8). The release profile of pure valsartan was compared to that from two different solid dispersion formulations. At any pH, the drug release was faster in the solid dispersions than in the pure form. The best and most complete drug release was achieved at a pH of 6.8; in these conditions, drug release from solid dispersions was considerably higher than that from pure drug. In vivo pharmacokinetic studies in rats confirmed the in vitro results, showing that the solid dispersion formulation had better pharmacokinetic parameters than the pure drug. The authors correlated the complete drug amorphization in the solid dispersion formulations, proven by FTIR, XRPD and DSC, to the increase in valsartan biovailability when prepared in these forms. 

Drug particle dissolution in extrudates was also examined in a study on the poorly soluble drug 17*β*-estradiol hemihydrate, extruded in presence of different polymers such as polyethylen glycol (PEG) 6000, Kollidon^®^ K30, and Kollidon^®^ VA64. Solid dispersions showed faster dissolution rates than pure crystalline drug or physical mixes [92]. 

In another such study, indomethacin (IDM) Form I was extruded with Eudragit^®^ RD 100 giving rise an amorphous mix. The extrusion promoted the formation of a solid solution with a continuous matrix structure responsible for the diffusion-controlled release of the drug from the extrudates [93].

In addition to the solid state transition of the drug during HME, the high shear stress associated with this method promotes the dispersion of the drug within the polymer, increasing drug–polymer interactions [94], and thus drug solubility and dissolution. 

For example, celecoxib was extruded with Eudragit 4155F, forming an amorphous solid dispersion with better bioavailability than that of the pure form. NMR proved an interaction between the drug and polymer that explained the increase in drug solubility [95]. 

In another study, glass solutions containing celecoxib and PVP were prepared by HME. The drug-polymer interactions were proven by the Gordon-Taylor equation and confirmed using FTIR and Raman spectroscopy. An increase in drug solubility was attributed to the formation of the glass solution and to the chemical interaction between drug and polymer [96].

Strong intermolecular interactions formed between drug and polymer were also fundamental in the study by Andrews et al. [97], who used HME to prepare solid molecular dispersions of bicalutamide and PVP by HME, and found that the drug dissolution rate of extrudates was significantly higher than that of crystalline drug and physical mixtures. They correlated this finding, proven by Gordon-Taylor equation and confirmed by FTIR and Raman spectroscopy, to the strong intermolecular interactions formed between drug and polymer. 

Drug-polymer interactions were important in a study on ibuprofen, hot melt extruded with Eudragit^®^ E PO to form an amorphous solid dispersion that exhibited excellent dissolution rate. FTIR confirmed this was due to ionic interactions between the carboxylic group of ibuprofen and dimethyl amino group of Eudragit^®^ E PO [98]. 

Similarly, FTIR spectroscopic imaging was successfully applied to investigate a drug dissolution process because this technique enables the generation of images in situ that reflect the spatial distribution of each formulation component, in particular the drug and polymer [99].

Starting from this premise, Pudlas and co-authors [100] applied this technique to evaluate the impact of the ibuprofen form and polymers (copovidone or Soluplus^®^) on particle dissolution. The IR signal was recovered directly from the dissolution cell operating in a continuous mode, while also recording the UV signal for the drug quantitation. FTIR images were separately selected at specific wavelength intervals for drug and polymers, and coloured images highlighted changes in concentration of drug with time. Results showed that drug release was influenced by both the polymer type and drug form, salt or acid. The salt ibuprofen did not interact with polymers, while the acidic form could form H-bonds with the polymers. The interaction reduced the drug release because polymer sites, generally involved in bonds with water, are saturated by the drug; faster drug release was observed in presence of copovidone because of its higher hydrophilicity. 

Another interesting aspect of drug-polymer interactions is the hydrophilicity of the polymer, which promotes the diffusion of water through the polymeric matrix, favouring contact between the drug and the water, and thus its dissolution [40,41].

Hydrophilic carriers such as PVP and PVP/VA, PEG, PEO, some celluloses, and polymethacrylate derivatives have been successfully used to enhance solubility and thus bioavailability of poorly soluble drugs using HME techniques [101].

Polymeric carriers can favour particle dissolution because they are able to separate solid particles and thus hinder their aggregation. For example, micronized particles of amorphous itraconazole were extruded in presence of poloxamer 407 and PEO 200 M. It was proved that particles disaggregate and disperse into the hydrophilic polymeric matrix, thus explaining the gain in drug delivery [102]. 

Drug release of theophilline extrudate in a polymer matrix was mostly controlled by drug loading during the first hour dissolution, but during the later time points it was controlled by the polymer and in minimal part by the process parameters [38]. 

The dissolution behaviour of an extrudate of itraconazole was compared to two amorphous solid dispersions. Results showed that the dissolution behaviour of the three formulations was strongly affected by the dissolution conditions and by the presence of surfactants that may influence the interaction between the formulation and the dissolution media [103]. 

The increase in drug release rate and bioavailability of two poorly soluble drugs, hydrochlorthiazide and celecoxib, was possible under HME with partially hydrolized polyvinyl alcohol with various degree of hydroxylation. The highest drug release rate was obtained with polyvinyl alcohol of the highest degree of substitution, because release was not affected by the ionic strength [39]. 

The particle dissolution profile of fenofibrate solid lipid nanoparticles (SLN), prepared with an innovative combination between HME and high pressure homogenization (HPH), revealed greater dissolution percentage in comparison with pure fenofibrate and a commercial formulation. In vitro data were confirmed by in vivo results showing that the innovative formulation had a better average plasma concentration time profile than the others. The authors optimized the experimental conditions for the preparation of the SLN using a Quality by Design approach. A Plackett-Burman design was thus adopted and response parameters were the mean particle size, polydispersity index and zeta potential determined by dynamic light scattering (DLS), and the drug encapsulation efficiency determined by HPLC-DAD. The authors concluded by highlighting the possibility of combining HME with another continuous process mode such as HPH to obtain a final formulation with optimized properties [21]. 

## 2. Conclusions

Hot Melt Extrusion is a tried and tested process widely used in the pharmaceutical industry, thanks to its many advantages, as demonstrated by the increasing number of pharmaceutical drug products produced by HME that are authorized for marketing. 3D printing, a natural evolution of HME, as it shares with HME the extrusion process, is now attracting increased interest. Growth in the number of authorized drug products produced using 3D printing should be expected in the coming years. 

While designing and optimizing an HME process, researchers and developers must take into account a variety of factors, such as the chemical and thermal degradation of the mix, the physicochemical stability of extrudates, the interaction of the drug with the other components of the mix, and the improvement in drug performance, for example, an increase in bioavailability.

**Figure 1:**
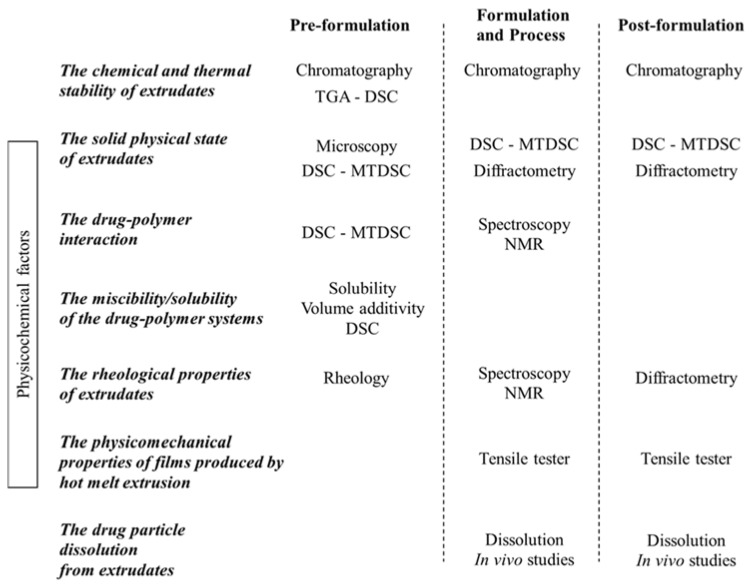
Techniques that can be applied during the different phases of Hot Melt Extrusion (HME), taking into account factors that should be investigated while designing and optimizing a Hot Melt Extrusion process.

Science and technology offer many solutions to respond to the needs of formulators. Through well-considered combinations of methodologies and approaches, formulators can optimize process parameters to achieve their specific objectives (Figure 1). 

As shown in this review, preliminary and quick studies combined with in-line process control technologies are successfully applied to optimize the hot melt extrusion process. The success of these technologies may involve an appropriate Design of Experiments (DoE) and PAT.

## Figures and Tables

**Table 1:** Advantages and disadvantage of Hot Melt Extrusion (HME) process.

Advantages	Explanation	References
Absence of solvents	This reduces the risk of chemical degradation of drug (i.e., hydrolysis in presence of water). This avoids residual organic solvents that may impact on the toxicity of the formulation. This can be considered a green technology.	[30,31,32]
Improved bioavailability	The improved solubility and bioavailability of poorly soluble drugs is one of the most frequently reasons for HME.	[13,14,15,16,30,31,32,33,34]
Modify drug release	The possibility to mix a drug with a polymer with specific solubility allows modification of the drug release (retarded, modulated, sustained).	[4,17,18,35,36,37,38,39,40,41]
Uniform dispersion of disperse solids in the molten mass	The uniform dispersion of the drug in a molten mass improves the drug homogeneity in the final dosage form.	[42,43,44,45,46,47]
Few processing steps	In general, few processing steps are necessary: classically, mixing, melting, solidification while extrusion, and downstream processes.	[1,26,44,48,49]
Continuous operation and ease of scalability	HME is classically a technique that operates in continuous mode. The HME process can be easily followed by Process Analytical Techniques (PAT) and several methods have been developed for this aim or for adaptation to HME.	[27,28,43,50]
No requirements for the compressibility of active ingredients	As in granulation technology, for active ingredients, flowability and compressibility are not required.	[4,51]
Wide range of dosage forms and delivery routes	Many dosage forms can be prepared by HME, such as granules, pellets, tablets, implants, and they can be delivered through various administration routes: oral, transmucosal, intradermal.	[3,4,5,6,7,8,9,10]
High process temperatures are necessary	High temperatures are necessary to promote the mix melt, which must be stable under thermal degradation.	[1,52]
High energy input coming from the applied shear forces	All the components, particularly polymers, must be stable under shear forces and not degraded under shear stresses.	[18,44,53]
Feedstock must have good flow properties	The mix that melts under extrusion must have good flow properties through the extrusion chamber.	[23,43]
Need for excipients	In addition to polymer and drug, other substances are necessary to increase the flow, or to modify the glass transition temperature, for example, plasticizers.	[25,54,55]

**Table 2:** Factors to be investigated while designing and optimizing a hot melt extrusion process: techniques used and explanation for their contribution.

Scope	Techniques	Technique Principle	Results	References
The chemical and thermal stability of extrudates	Chromatographic techniques High Performance Liquid Chromatography, HPLC High Performance Liquid Chromatography Mass Spectrometry, HPLC-MS High Performance Liquid Chromatography Photo Diode Array, HPLC-PDA Gas-Chromatography Mass Spectrometry, GC-MS Gel Permeation Chomatography, GPC	Chromatographic techniques are based on the separation of different analytes from complex mixtures based upon several factors (solubility/miscibility, column affinity, volatility, molecular weight, etc.). Analytes can be detected by different detectors (i.e., mass spectrometer, photo diode array, etc.).	Separate/identify different analytes in the mix. Evaluate chemical and thermal stability of drugs and mixes. Evaluate chemical and thermal stability of polymers. Identify degradation products. Describe degradation mechanism.	[4,14,18,30,31,44,56,57,58,59,60,61,62]
	Thermal analysis techniques Themogravimetry, TGA Differential Scanning Calorimetry, DSC Modulated Differential Scanning Calorimetry, MDSC	Thermal analysis techniques are based on isothermal, scanning, and modulated temperature. A change in weight or heat flow of the samples is recorded.	Identification of sample thermal degradation. Assessment of thermal stability of drug and mixes. Identification of the temperature interval for the HME process.	[4,14,32,44,45,49,52,59,63,64]
The solid physical state of extrudates	Hot stage microscopy (HSM) Hot stage polarized light microscopy (HS-PLM) Atomic force microscopy (AFM)	Microscopy techniques combine information from microscopy and physical state of the sample. HSM combines results from microscopy and thermal analysis. HS-PLM combines results from microscopy and thermal analysis under polarized light. AFM combines results from microscopy and solid state.	Evaluation of changes in particle size, particle morphology, and solid physical state under heating. Evaluation of the solid physical state (amorphous, crystalline or partially amorphous) of extrudates.	[14,38,44,59,65]
	X-ray Powder Diffractometry (XRPD)	X-ray beams hitting crystalline solid materials are scattered in all directions, producing distinct scattering patterns, similar to fingerprints. A halo, that is the absence of diffraction peaks, corresponds to a completely amorphous sample. The crystallinity degree can be calculated by Ruland’s or Hermans and Weidinger’s methods.	Evaluation of changes in crystallinity degree. Amorphization. Presence of physical mixtures, solid dispersions, or solid solutions.	[1,38,44,54,61,66,67,68,69,70,71]
	Thermal analysis techniques Themogravimetry, TGA Differential Scanning Calorimetry, DSC Modulated Differential Scanning Calorimetry, MDSC	See above in the table	Determination of solid state transitions. Determination of glass transition, melting temperatures, changes in weight. Determination of crystalline degree. Crystallization tendency. Tendency to amorphization.	[14,54,64,68,71,72,73]
The drug–polymer interaction	Spectroscopic techniques Fourier-transform infrared (FTIR) spectroscopy Attenuated total reflectance-Fourier transform infrared (ATR-FTIR) spectroscopy X-ray photon spectroscopy (XPS) Raman spectroscopy Nuclear magnetic resonance (NMR)	Spectroscopic techniques are based on the exposure of molecules to different radiations (vibrational, magnetic, …). Information can be qualitative and quantitative.	Identification of interaction between molecules.	[10,38,44,50,52,61,74,75,76,77,78]
The miscibility/solubility of drug–polymer systems	Solubility parameter (Hansen’s Method) Measurement of glass transition temperature (for example by DSC). Theoretical prediction of glass transition temperature based on the volume additivity of a mix (from the Gordon Taylor equation)	The miscibility drug–polymer can be deduced by solubility parameters. The solubility parameter can predict if one material will dissolve in another to form a solution. Thus, it can be used to predict miscibility of drugs and excipients. The miscibility/solubility of drug–polymer system has important repercussions on feasibility process and increase in dissolution rate. The glass transition temperature can be theoretically predicted from the Gordon Taylor equation. Deviation from the predicted values can be explained in term of immiscibility or in term of enhanced molecule-molecule interaction.	The ability of a drug to dissolve in binary, ternary, complex mix. The evaluation of formation of solid dispersions, solid solutions, physical mixes.	[39,54,55,59,74,79,80,81,82,83,84]
The rheological properties of extrudates	Rheology (oscillatory rheometer, torque rheometer)	By applying a shear stress or a shear strain on a free flowing material, rheology permits to characterize the flowing properties of a material, according to the temperature.	The evaluation of viscoelastic properties of a material subjected to HME for predicting the process feasibility. The measurement of viscosity. The prediction of the rheological behavior of polymers under heating and extrusion. The selection of the most appropriate formulation (for example drug–polymer ratio, the necessity for the addition of plasticizers) and the best operating conditions (temperature).	[29,32,52,63,74,85,86]
Physicomechanical properties of films produced by hot melt extrusion	Tensile tester	The tensile tester permits the characterization of films produced by the HME process. Several parameters can be determined such as the elastic modulus, the tensile strength and the elongation.		[33,53,87,88,89]
The drug particle dissolution from extrudates	Dissolution testing (according to different Pharmacopeias)	Well standardized apparatus and methods are described by different Pharmacopeias. The test is based on the dissolution of drug molecules in a dissolution medium during a specific time interval under validated conditions. The drug dissolved can be quantified by different methods (UV, MS). Results can be confirmed by different in vivo methods.	The amount of drug dissolved with time. The drug dissolution profile. The evaluation of mechanisms for drug release (zero order, first order release mechanisms).	[21,34,38,39,40,41,90,91,92,93,94,95,96,97,98,99,100,101,102,103]
